# Unraveling of Poly(lactic acid) (PLA)/Natural Wax/Titanium Dioxide Nanoparticle Composites for Disposable Plastic Applications

**DOI:** 10.3390/polym17050685

**Published:** 2025-03-04

**Authors:** Jacqueline Guadalupe Bocarando-Chacón, Iván Alziri Estrada-Moreno, Imelda Olivas-Armendáriz, Alejandro Vega-Rios, Mónica Elvira Mendoza-Duarte

**Affiliations:** 1 División Industrial, Universidad Tecnológica de Querétaro, Av. Pie de la Cuesta 2501, Nacional, Qro, Santiago de Querétaro 76148, Mexico; jacqueline.bocarando@uteq.edu.mx; 2 Secretaría de Ciencia, Humanidades, Tecnología e Innovación (SECIHTI)—Centro de Investigación en Materiales Avanzados, S.C. (CIMAV), Av. Miguel de Cervantes #120, Chihuahua 31136, Mexico; ivan.estrada@cimav.edu.mx; 3 Institute of Engineering and Technology, Autonomous University of the City of Juárez (UACJ), Ave. Del Charro 450 Norte, Ciudad Juárez 32310, Mexico; iolivas@uacj.mx; 4 Centro de Investigación en Materiales Avanzados, S.C. (CIMAV), Av. Miguel de Cervantes #120, Chihuahua 31136, Mexico

**Keywords:** PLA, natural wax, TiO_2_ nanoparticles, antibacterial activity, hydrolytic degradation, optical transparency

## Abstract

The present research is a comprehensive study that developed poly(lactic acid) PLA/natural wax (Wx)/non-functionalized titanium dioxide nanoparticles (TiO_2_-NF) and PLA/Wx/titanium dioxide nanoparticles functionalized with triethoxysilane (TiO_2_-F) composites by melt blending. This research systematically investigated their hydrolytic degradation, antibacterial properties, oxygen permeability, and optical transparency. The TiO_2_-NF or TiO_2_-F (0.1, 0.5, or 1 wt%) were added to a PLA/Wx (85:15) blend using a Brabender internal mixer at 180 °C. Hydrolytic degradation was carried out in distilled water at 50 °C and an initial pH of 6.2 for 9 months. Changes in weight, morphology, and the rheological behavior of the blends were evaluated at different times during the hydrolytic degradation of the PLA/Wx/TiO_2_-NF and PLA/Wx/TiO_2_-F composites. The antibacterial properties of PLA/Wx, PLA/Wx/TiO_2_-0.1-NF, and PLA/Wx/TiO_2_-0.1-F were assessed by testing them against both *E. coli* (Gram-negative) and *S. aureus* (Gram-positive) bacteria. Their oxygen permeability and optical transparency are comparable to those of LDPE films. These composites, produced by melt blending, show potential for application as disposable plastics, which could significantly impact the fields of materials science and polymer engineering.

## 1. Introduction

The surge in demand for single-use products has led to numerous environmental issues, which are driven by significant pollution and waste management challenges. The accumulation of plastic waste in the environment has become a critical global issue with far-reaching ecological, economic, and social consequences. In response to this enormous challenge, biodegradable polymers have garnered considerable attention due to their potential to mitigate long-term environmental pollution. The ability to biodegrade has thus become a crucial aspect when developing one-time-use polymer materials, particularly for applications such as food packaging [1]. Among these materials, poly(lactic acid) (PLA) stands out as one of the most promising alternatives to conventional petroleum-based plastics [2].

PLA is a biodegradable thermoplastic derived from renewable resources such as corn starch, maize, sugarcane, or milk-derived lactic acid. This environmentally friendly material combines biodegradability and sustainability, making it an attractive alternative to traditional plastics [2]. PLA has a similar appearance to clear polystyrene, with high gloss and transparency. However, it has several drawbacks, including low mechanical properties (rigidity and fragility) and low thermal resistance, and often requires the incorporation of plasticizers to improve its flexibility. Additionally, PLA has a slow degradation rate under certain conditions, limiting its use, especially in single-use products [3].

Several strategies have been employed to overcome the aforementioned drawbacks of PLA, including polymer blends, plasticizers, fillers, and additives such as metallic oxides. Despite these efforts, few studies have focused on the application of PLA in combination with natural waxes (Wx), which act as effective plasticizers, enhancing the flexibility and processability of PLA while preserving its biodegradability. For example, Mendoza-Duarte M. et al. demonstrated that mixing natural waxes, e.g., beeswax or cocoa, with PLA at 15 wt% concentrations results in a favorable balance of mechanical properties [1].

Moreover, metallic oxides have been extensively used as additives in biopolymers used for food packaging because of their strong antibacterial properties and ability to facilitate ethylene photolysis [4]. Likewise, the incorporation of nanoparticles, e.g., titanium dioxide (TiO_2_), has improved the mechanical, thermal, and photodegradation properties of PLA composites [5,6,7]. For instance, TiO_2_ with sizes ranging from the nanoparticle to macroparticle has several advantages, such as being cost-effective, highly thermostable, photocatalytically active, capable of absorbing ultraviolet light and having low toxicity, antibacterial properties, and adequate biocompatibility [4,8,9,10,11]. In order to enhance specific properties, advanced materials are often tailored by functionalization [12]. For instance, metal–organic frameworks, including ZIF-67-based materials, are modified via doping, hybridization, and surface functionalization to improve their adsorption efficiency and selectivity in environmental applications. Typically, methods used for the surface functionalization of TiO_2_ nanoparticles involve a two-stage approach. First, the nanoparticles are synthesized, and then a subsequent post-functionalization step is carried out to modify their surface properties [13].

Furthermore, TiO_2_ has been approved as a food additive due to the low toxicity, up to a concentration of 1000 mg/Kg body weight, of TiO_2_ nanoparticles smaller than 30 nm. However, when TiO_2_ nanoparticles (>30 nm) are utilized, the highest dose tested is 100 mg/Kg body weight, which is the value published by the European Food Safety Authority (EFSA) based on their safety assessment of TiO_2_ as a food additive [14]. In previous studies, such as those conducted by Li Wenhui et al. [15], the migration of PLA/TiO_2_ nanoparticles (3 wt%) was reported to be 112.92 μg/kg. However, even when the potential to enhance performance through composite materials has been explored in recent studies, the combination of PLA with natural waxes and TiO_2_ nanoparticles remains underexplored.

Due to the massive employment of plastics for food packaging, it is essential to find an alternative to quickly decompose materials [16]. In addition, when thinking about employing a material in food packaging products, it is necessary to control the exchange of gases between the inside and outside of the package by regulating the packaging’s gas permeability (GP), as this plays a crucial role in prolonging the shelf life of food products [17].

Moreover, following a bulk erosion process, PLA degrades through hydrolytic cleavage [3]. Hydrolytic degradation is a key mechanism behind the breakdown of polylactic acid (PLA) in aqueous conditions. PLA undergoes hydrolytic degradation primarily through chain scission facilitated by water, temperature, and pH, and the presence of additives or fillers can significantly influence this degradation [18,19]. Chain scission primarily occurs in amorphous regions, enabling its carboxylic end groups to act catalytically, accelerating the hydrolytic degradation of PLA in a self-catalyzed process [19,20,21,22]. Although there has been extensive research on PLA degradation, there is still a need to investigate the combined effects of plasticizers and nanoparticles on its hydrolytic behavior, particularly under conditions that mimic real-world environments. The presence of plasticizers and nanoparticles can influence the degradation process, making it essential to explore the combined effects of these additives.

The present study aims to address this gap in the literature by investigating the hydrolytic degradation, antibacterial activity, oxygen permeability, and optical transparency of PLA/natural wax (Wx)/non-functionalized TiO_2_ nanoparticles (TiO_2_-NF) and PLA/natural wax (Wx)/TiO_2_ nanoparticles functionalized with triethoxysilane (TiO_2_-F). Furthermore, TiO_2_-NF and TiO_2_-F were employed at concentrations of 0.1, 0.5, and 1.0 wt%. The rheological properties, surface morphology, water absorption, and weight changes observed during degradation were studied in distilled water (with an initial pH of 6.2 ± 0.3) at 50 °C for 9 months. This study offers a comprehensive analysis of PLA/Wx/TiO_2_-NF and PLA/Wx/TiO_2_-F composites for enhancing the development of PLA-based materials with improved performance and customized degradation profiles for sustainable applications, such as food containers. The findings of this study could significantly contribute to the advancement of materials science and the food packaging industry by providing a deeper understanding of the potential of PLA composites for sustainable food packaging.

## 2. Materials and Methods

### 2.1. Materials

Poly (L,D—lactide) PLA Ingeo 4060D, (8–10%) D was sourced from NatureWorks LLC, (Plymouth, MN, USA), with an average molecular weight Mw of −190 kg/mol, ρ = 1.24 g/cm^3^, and a glass transition temperature Tg = (55–60) °C. Natural waxes: beeswax (BW) and cocoa (CCA) were provided by Reactivos de Laboratorio (Requilab, Chihuahua, Chihuahua, Mexico), both with ρ = 0.98 g/cm^3^ at 20 °C, and were used as received. Lactic Acid, 85% ACS (Fermont), was employed as a reference for the FTIR analysis. Titanium (IV) isopropoxide (C_12_H_28_O_4_Ti), ethanol (C_2_H_6_O), distilled water, heptane, and triethoxysilane were provided by Reactivos Química Meyer (Cd. Mexico, Mexico).

### 2.2. Synthesis of Non-Functionalized Titanium Dioxide Nanoparticles

The sol–gel method was employed to synthesize the non-functionalized titanium dioxide nanoparticles, namely TiO_2_-NF. First, 5 mL of titanium (IV) isopropoxide was mixed (stirred vigorously) in 300 mL of ethanol until a homogeneous solution formed. Subsequently, 50 mL of distilled water was dripped into the titanium (IV) isopropoxide–ethanol solution. The addition of water caused the hydrolysis of titanium (IV) isopropoxide, forming hydroxyl groups (-OH) and releasing isopropanol. The obtained precipitate was filtered and washed with distilled water to remove impurities and dried at a temperature of 60 °C for 24 h in a drying oven. The resulting powder was subjected to a calcination treatment at 550 °C for 2 h to obtain the desired crystalline structure.

### 2.3. Synthesis of Functionalized Titanium Dioxide Nanoparticles

The synthesis of the functionalized titanium dioxide nanoparticles (TiO_2_-F) was performed through the dispersion of TiO_2_-NF in 50 mL of heptane employing a Cole-Parmer ultrasonic bath, model 8893 (Vernon Hills, IL, USA). Next, 5 mL of triethoxysilane was dripped into the heptane and TiO_2_-NF dispersion for 30 min at a temperature of 50 °C. Finally, the product (TiO_2_-F) was filtrated and washed three times with heptane.

### 2.4. Formulation of PLA/Natural Wax/TiO_2_-NF and PLA/Natural Wax/TiO_2_-F Composites

Prior to melt blending, the PLA underwent a drying process to remove moisture in a Fisherbrand Isotemp Vaccum Oven, model 281A (Pittsburgh, PA, USA) at 60 °C for 12 h. Meanwhile, the TiO_2_-NF or TiO_2_-F and Wx (with a 50/50 ratio of BW/CCA) were placed in a beaker on a hot plate under magnetic stirring at a temperature of 70 °C for 10 min..

The blending process was conducted using a Brabender internal mixer (BB), model DDRV501 (C.W. Brabender Instruments, Inc., Hackensack, NJ, USA) operating at a temperature of 180 °C for 7 min and employing CAM-type blades. The blending procedure involved a programmed speed of 30 rpm for the first 2 min, followed by an increase to 50 rpm for the remaining 5 min. Likewise, as blank, a formulation was prepared containing neat PLA and the two waxes, as described in the methodology in our previous investigation [1]. The formulations of the polymer blends produced are presented in Table 1. Our study selected the TiO_2_-NF or TiO_2_-F concentrations that would prevent particle migration in their final application [15].

#### Preparation of Films and Probes

The obtained blends were cooled to ambient temperature and processed using a blade mill, Fritsch model Pulverisette (Idar-Oberstein, Germany). In order to evaluate the UV-Vis transparency and the hydrolytic degradation of the blends, thin films (with a thickness of 0.15 mm) and type V probes (ASTM D638) [23] were obtained, respectively. A 5.5 g sample of pellets was used to obtain a thin film using a hydraulic compression molding press (Carver Inc., Wabash, IN, USA) at a temperature of 180 °C. First, the pellets were put between two metal plates and softened for 2 min without force; subsequently, for films and type V probes a force of 1 metric ton (mt) and 3 mt was applied for 4 min, respectively. The material was rapidly cooled to 12 °C using a water cooling bath and left for 5 min.

### 2.5. Hydrolytic Degradation

The experiments were carried out in tridistilled water (pH = 6.2 ± 0.3) at a temperature of 50.0 ± 0.1 °C, close to the glass transition temperature of PLA, and in an acid medium in order to accelerate the PLA’s hydrolytic degradation. Seven probes for each formulation (ASTM D638 Type V) [23] were vertically positioned inside acrylic boxes. Water (2 L) was then added, covering the 1 cm above the samples. Furthermore, one specimen from each formulation was extracted from the boxes at intervals of 7, 21, 42, 63, 105, 168, and 275 days for examination. The acrylic boxes containing the probes were kept in a forced-air oven at a constant temperature of 50.0 °C for 275 days. The water in each sample remained unchanged throughout the experiment.

### 2.6. Characterization of TiO_2_-NF and TiO_2_-F Nanoparticles

#### 2.6.1. Morphology

STEM images were captured using a Transmission Electron Microscope, JEOL TM JEM2200 CS/UHR, (Tokyo, Japan) with Bright Field (BF) detector model AZtecLive Ultim Max (Oxford, UK). The applied voltage was 200.00 kV. The samples were prepared on a Formvar/carbon 300-mesh copper grid.

#### 2.6.2. X-Ray Diffraction

The XRD data were collected using a Rigaku X-ray diffractometer Model Miniflex, (Tokyo, Japan) that used CuKα (30 kV, 15 mA, λ = 1.5406 Å). The 2θ diffraction diagrams were recorded from 5° to 80° with a step of 2° min^−1^.

#### 2.6.3. FTIR

The structural characterization of the samples was carried out using Fourier Transform Infrared Spectroscopy (FTIR). FTIR spectra were obtained using an IR Afinity 1S B spectrometer (Shimadzu, Kyoto, Japan) equipped with a Total Attenuated Reflectance accessory, Smiths model Quest. The samples were analyzed in transmittance mode within the range of 600 cm^−1^ to 4000 cm^−1^ with a resolution of 4 cm^−1^.

### 2.7. Characterization of PLA/Natural Wax/TiO_2_-NF and PLA/Natural Wax/TiO_2_-F Composites

#### 2.7.1. Chemical Structure

The samples were analyzed under the same FTIR conditions as the TiO_2_-NF and TiO_2_-F; see Section 2.6.3.

#### 2.7.2. Water Absorption and Remaining Mass

The initial weight (W_i_) of the samples was registered prior to hydrolytic degradation using a 4-digit analytical balance OHAUS Adventurer AR3130, (Ohaus Corporation, NJ, USA). Subsequently, the weight of three samples at each specific time (0, 7, 21, 42, 63, 105, and 168 days) was registered. Then, samples were weighed to determine their wet weight (W_w_) by removing excess water with Kimwipes Kimtech science, (Kimberly-Clark Corporation, Dallas, TX, USA). Later, these probes were rinsed with distilled water and dried at 25 °C for 72 h at a set vacuum of 0.8 bar until a constant dry weight (W_absorption_). Finally, the percentage of water absorption (W_absoprtion_) seen and the remaining mass (R_mass_) were determined according to Equation (1) [19] and Equation (2) [19], respectively.

W_absorption_ (wt%) = (W_w_ − W_d_)/(W_d_) × 100 (1)

R_mass_ (wt%) = 100 − Weight loss (%) = (W_d_)/(W_i_) × 100 (2)

#### 2.7.3. pH

The pH value of the solution was measured using a pH meter (Thermosicentific Eutech Elite pH, Singapore) at the previously designated periods.

#### 2.7.4. Scanning Electron Microscopy (SEM)

The PLA and its blends were examined using a scanning electron microscope, model Hitachi SU3500 (Tokyo, Japan), operating at 5.00 kV. Before analysis, the samples were coated with a fine layer of gold to enhance their conductivity.

#### 2.7.5. Melt Rheology

The rheological behavior of the PLA/Wax/TiO_2_ (NF and F nanoparticles) blends was evaluated using a rotational rheometer, a Physica MCR 501 (Anton Paar, Graz, Austria). Due to the nature of the samples, and in order to obtain graphs without noise, three different strain levels were applied, instead of using a single strain for the entire frequency sweep, as follows: from 100 Hz to 1.47 Hz, the applied strain was 0.001%; from 1 Hz to 0.14 Hz, the applied strain was 0.01%; and from 0.1 Hz to 0.01 Hz, the applied strain was 0.1%. All applied strains were chosen to be within the linear viscoelastic region by conducting a preliminary strain sweep test. Tests were carried out at a temperature of 170 °C, employing a 40 mm diameter plate–plate geometry.

#### 2.7.6. Antibacterial Activity

In this experiment, all samples (8 mm in diameter) were sterilized using UV light inside a laminar flow hood (LABCONCO) for 15 min on each side before testing. Under sterile conditions, standard solutions containing approximately 1.5 × 10^8^ CFU/mL of Escherichia coli (ATCC^®^ 11229™) and Staphylococcus aureus (ATCC^®^ 6538™) were prepared for testing. These bacteria were then inoculated onto separate Petri dishes containing nutrient agar (Difco 21300). The samples were subsequently placed onto the inoculated agar plates. The Petri dishes were incubated at 37 °C for 24 and 48 h, after which inhibition zones were observed and measured using ImageJ 1.54g software. The analysis was carried out in triplicate. The data collected were analyzed using the data analysis tool in Microsoft Excel. In order to examine the means across groups, Student’s *t*-test was applied, with a significance level of *p* < 0.01.

#### 2.7.7. Oxygen Gas Transmission Rate

To determine the steady-state rate of oxygen gas transmission through the PLA/Wx/TiO_2_-NF and PLA/Wx/TiO_2_-F composites in film form, ASTM D3985-95 “Oxygen Gas Transmission Rate Through Plastic Film and Sheeting Using a Coloumetric Sensor” was followed [24]. The employed equipment was an OX-TRAN 2/61 Oxygen Permeability, model 2/61 (Mocon, Inc., Minneapolis, MN, USA).

#### 2.7.8. Optical Transparency

The typical method used to characterize optical transparency is transmittance. An Evolution 220 UV–visible spectrophotometer (Waltham, MA, USA) was utilized. The spectra of the PLA/Wx/TiO_2_-NF and PLA/Wx/TiO_2_-F composites in film form were captured using wavelengths between 200 and 900 nm.

## 3. Results and Discussion

**Figure 1:**
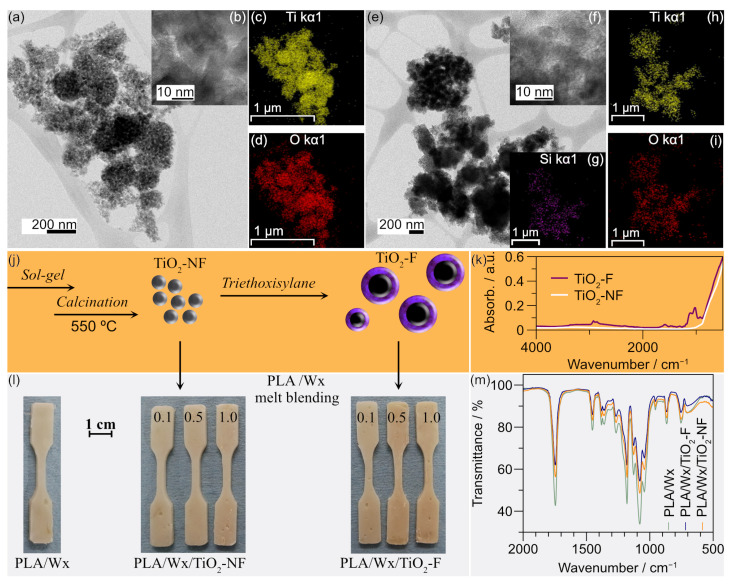
STEM micrographs. (**a**) Non-functionalized TiO_2_ nanoparticles (TiO_2_-NF). (**b**) TiO_2_-NF magnification at 10 nm. (**c**,**d**) Elemental mapping of Ti and O, respectively; (**e**) TiO_2_ nanoparticles functionalized with triethoxysilane (TiO_2_-F). (**f**) TiO_2_-NF magnification at 10 nm. (**g**–**i**) Elemental mapping of Si, Ti, and O, respectively. (**j**) Scheme of TiO_2_-NF and TiO_2_-F synthesis. (**k**) FTIR spectra of TiO_2_-NF and TiO_2_-F. (**l**) Photos of polylactic acid/natural waxes (PLA/Wx), PLA/Wx/non-functionalized TiO_2_ nanoparticles (PLA/Wx/TiO_2_-NF), and PLA/Wx/TiO_2_ nanoparticles functionalized with triethoxysilane (PLA/Wx/TiO_2_-F) probes. (**m**) FTIR spectra of PLA/Wx, PLA/Wx/TiO_2_-NF, and PLA/Wx/TiO_2_-F.

The morphology of the titanium dioxide nanoparticles functionalized with triethoxysilane (TiO_2_-F) and those that were non-functionalized (TiO_2_-NF) was characterized by STEM; see Figure 1. Figure 1a displays the micrographs of TiO_2_-NF, where the nanoparticles are round and of different sizes. Figure 1b illustrates the magnification of TiO_2_-NF. The particle size distribution of TiO_2_-NF is from 11 nm to 37 nm, Figure S1. This heterogeneity is due to the presence of water during the hydrolysis and condensation reactions, which occur expeditiously. Consequently, the different shapes and sizes of the nanoparticles are poor [25]. The elemental mapping of TiO_2_-NF reveals the existence of Ti and O; see Figure 1c and Figure 1d, respectively. The elemental composition identified by the energy-dispersive X-ray (EDX) analysis confirms the chemical structure of the TiO_2_ as being 67% atomic O and 32% atomic Ti; see Figure S2. Furthermore, the XRD spectrum of TiO_2_-NF confirms it has an anatase-type phase; see Figure S3.

Moreover, the micrograph of TiO_2_-F (Figure 1e) illustrates the presence of agglomerated nanoparticles due to the sample preparation method, which utilized isopropyl alcohol as a solvent. Figure 1f shows the magnification of TiO_2_-F; nevertheless, evidence of functionalization with triethoxysilane is unclear. The particle size distribution of TiO_2_-F is similar to that of TiO_2_-NF; see Figure S4. Liao et al. [26] reported a significant increase in TiO_2_ nanoparticles when they were functionalized with tetramethoxyl (octadecyl) silane. Therefore, it was necessary to perform an elemental mapping to check their functionalization. The elemental mapping of TiO_2_-F shows the presence of Si, Ti, and O, Figure 1g, 1h, and 1i, respectively. The EDX confirms that it contains 8.8% atomic silicon; see Figure S5.

Figure 1j illustrates the scheme used for the synthesis of TiO_2_-NF, including sol–gel and its functionalization with triethoxysilane to obtain TiO_2_-F. This functionalization was also corroborated by FTIR; see Figure 1k. Compared with TiO_2_-NF, the spectrum of TiO_2_-F exhibits molecular vibrations corresponding to the silane group (Si–H) from 1080 to 1100 cm^−1^, and also the presence of ethoxy group bands, and Si–O stretching close to 1100 cm^−1^ [27]. In addition, C–H stretching vibrations (2975–2840 cm^−1^), an asymmetric deformation band (1465–1440 cm^−1^), and a symmetric band (1390–1370 cm^−1^) corresponding to the methyl group or the methylene of triethoxysilane can also be identified [28].

Figure 1l illustrates photos of the polylactic acid/natural wax (PLA/Wx), PLA/Wx/non-functionalized titanium dioxide nanoparticle (PLA/Wx/TiO_2_-NF), and PLA/Wx/functionalized titanium dioxide nanoparticle (PLA/Wx/TiO_2_-F) composites. Figure 1m displays the FTIR spectra of the PLA/Wx, PLA/Wx/TiO_2_-NF, and PLA/Wx/TiO_2_-F. The FTIR spectra of PLA/Wx/TiO_2_-NF and PLA/Wx/TiO_2_-F were obtained using a 1 wt% of TiO_2_-NF or TiO_2_-F, i.e., PLA/Wx/TiO_2_-1.0-NF and PLA/Wx/TiO_2_-1.0-F, respectively. Compared to PLA/Wx, the FTIR spectra of PLA/Wx/TiO_2_-1.0-NF and PLA/Wx/TiO_2_-1.0-F show insignificant changes that correspond to new bands or shifts. This finding may be explained by the fact that the spectrum of the neat PLA (Figure S6) exhibits characteristic peaks close to 2950 and 2850 cm^−1^ which are associated with symmetric and asymmetric vibrations of the C–H bond. The peak at 1747 cm^−1^ is attributed to the presence of carbonyl groups, and the one at 1080 cm^−1^ corresponds to C–CO–C asymmetric stretching. Likewise, the Wx (beeswax and cocoa) spectra display similar molecular vibrations; see Figure S6. The wax spectrum displays peaks at 2900 and 2800 cm^−1^, indicating the presence of lipids. The peak at 1730 cm^−1^ is associated with unconjugated triglycerides, and the peak at 700 cm^−1^ corresponds to the vibration of the C=C bond, which is characteristic of fatty acids [29,30]. The main components of the composites have similar molecular vibrations, resulting in a spectrum without changes or shifts.

### 3.1. Water Absorption and Remaining Mass During Hydrolytic Degradation

The effects of the hydrolytic degradation on PLA/Wx/TiO_2_-NF and PLA/Wx/TiO_2_-F were studied. Their water absorption and remaining mass, which are closely linked to hydrolytic degradation, were tracked over 275 days (9 months). The probes were unable to be weighed after 275 days due to their complete disintegration and tendency to dissolve in the water medium. In Section 3.3, which details their scanning electron microscopy, we will discuss this.

**Figure 2:**
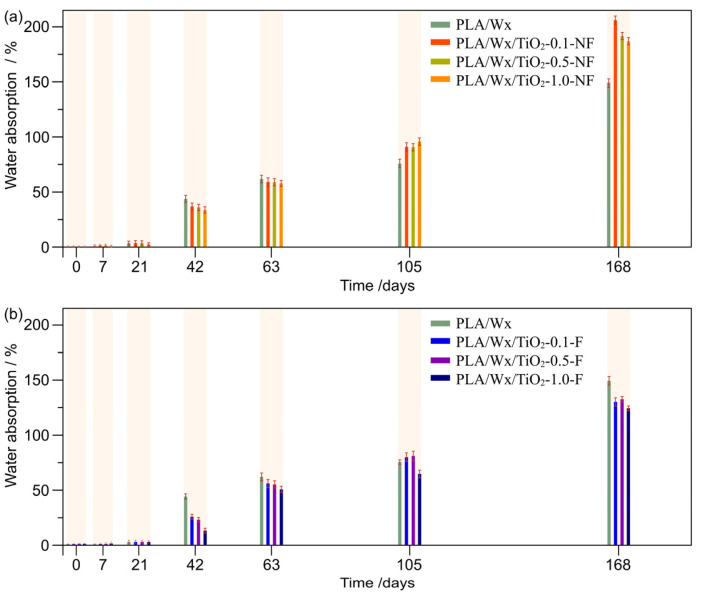
Water absorption study with regard to time. (**a**) PLA/Wx/TiO_2_-X-NF—where X = concentration, and in particular, 0.1, 0.5, or 1.0 wt%—vs. PLA/Wx; (**b**) PLA/Wx/TiO_2_-X-F—where X = concentration, and in particular, 0.1, 0.5, or 1.0 wt%.

Figure 2 illustrates the water absorption evolution seen during the hydrolytic degradation of the PLA/Wx/TiO_2_ blends. For the first 21 days, the water absorption remained minimal in all samples. Similar trends have been reported by Luo et al. [19], who observed that the water absorption in samples was relatively slow in the early stages, with a noticeable delay in water uptake. In the initial degradation period, the PLA matrix, which is hydrophobic, limits the amount of water that can diffuse into the material. This phenomenon would explain the minimal change in water absorption in the early days of our experiment. Moreover, the TiO_2_ nanofillers, although hydrophilic, are partially removed from direct contact with the water due to their distribution within the PLA matrix. The TiO_2_-NF and TiO_2_-F are more likely to remain in regions with limited water uptake, especially at lower filler concentrations, thus contributing to their slower water absorption rate. In our work, we compared low filler contents, which were distributed entirely into the polymer matrix; this may result in fewer sites for water absorption, thus leading to a minimal change in water uptake over the first 21 days.

Nevertheless, after 21 days, water absorption increases progressively with length of their exposure to water. This trend suggests that as the material undergoes degradation over time, its ability to absorb water is enhanced as a consequence of the breakdown of its structural integrity, which facilitates its substantial interaction with water molecules. This behavior is more evident in PLA/Wx/TiO_2_-NF samples than in PLA/Wx/TiO_2_-F blends. The above can be attributed to the fact that the TiO_2_-NF behaves solely as an inclusion within the PLA/Wx matrix. As the length of their exposure to water increases, the TiO_2_-NF nanoparticles detach, leading to a tremendous amount of water retention. At 168 days of hydrolytic degradation, significant water absorption is observed in the PLA/Wx/TiO_2_-NF samples compared to the PLA/Wx (149%), especially for PLA/Wx/TiO_2_-0.1-NF, which had a value of around 204%, Figure 2a.

On the other hand, the PLA/Wx/TiO_2_-F composites, as seen in Figure 2b, inhibit the uptake of water in the PLA/Wx matrix due to their functionalization with silane. Compared with PLA/Wx, PLA/Wx/TiO_2_-F diminishes its water absorption by 17%.

**Figure 3:**
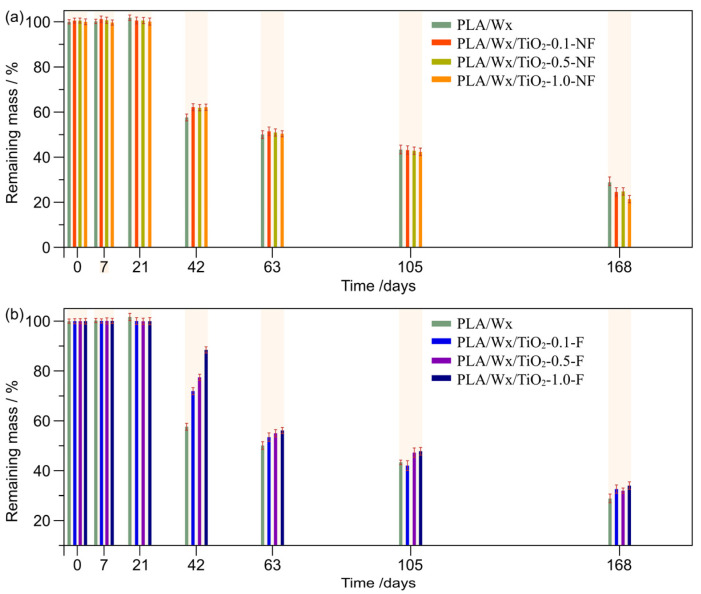
Study of remaining mass with regard to time. (**a**) PLA/Wx/TiO_2_-X-NF—X = concentration, in particular, 0.1, 0.5, or 1.0 wt%—vs. PLA/Wx; (**b**) PLA/Wx/TiO_2_-X-F—X = concentration, in particular, 0.1, 0.5, or 1.0 wt%.

The results for the remaining mass (R_mass_) of the PLA/Wx/TiO_2_ blends, based on Equation (2), during their hydrolytic degradation are shown in Figure 3. The differences between PLA/Wx/TiO_2_-NF and PLA/Wx/TiO_2_-F are insignificant until day 21, with a variation of ±1%. Nevertheless, at 40 days of hydrolytic degradation, all samples had a faster mass loss rate. The amount of R_mass_ in the PLA/Wax/TiO_2_-NF samples was significantly different than that of the PLA/Wax/TiO_2_-F blends, with mean values near 67% and 90%, respectively. In contrast, PLA/Wx registered an R_mass_ close to 60%. Therefore, these findings suggest that the hydrolytic degradation of the PLA/Wx matrix may be regulated by varying the concentration of TiO_2_-NF and TiO_2_-F nanoparticles within it, and this effect becomes more pronounced when the particles are chemically functionalized. However, in the scientific literature, systems of PLA/TiO_2_ composite films were found to degrade hydrolytically faster over short degradation times (in NaOH solution media at 37 °C) than neat PLA [31,32]. Nonetheless, in our study, the hydrolytic degradation occurred under different conditions, i.e., water at a neutral pH and a temperature of 50 °C.

On the other hand, once the samples had been submitted to the hydrolytic degradation test for 18 days, it was evident that the addition of TiO_2_-NF resulted in a significant weight loss compared to the control sample. This finding may be explained by the fact that the addition of TiO_2_-NF allows the liquid to permeate into the interior of the PLA/Wx matrix once the TiO_2_-NF detaches due to the lack of chemical functionalization to keep these nanoparticles strongly bound to the polymer. Therefore, at enhanced TiO_2_-NF concentrations, more liquid will penetrate into the matrix, leading to increased degradation.

Wang et al. [33] prepared PLA/TiO_2_ up-conversion nanoparticles, modified with Yb3+/Er3+/Tm3+ ions integrated into the TiO_2_ lattice and others adhered to the exterior of the TiO_2_ grains. They estimated the remaining weight of nanocomposites to be around 20–25% under exposure to solar radiation (>500 h) and air. Our study registered an insignificant weight loss at 20 days of hydrolytic degradation (with water at pH of 6.2 and at 50 °C), making this blend more stable. However, at 40 days, a weight loss of around 40% was measured for all samples.

### 3.2. pH Determined During Hydrolytic Degradation

**Figure 4:**
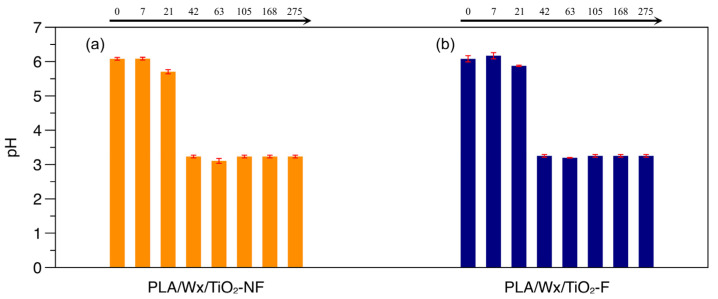
Evolution of pH during hydrolytic degradation. (**a**) PLA/Wx/TiO_2_-NF; (**b**) PLA/Wx/TiO_2_-F.

pH is an essential factor in the hydrolytic degradation of PLA because hydrolysis is catalyzed by acids and bases [34]. Figure 4a,b illustrate the behavior of pH during the hydrolytic degradation of PLA/Wx/TiO_2_-NF and PLA/Wx/TiO_2_-F, respectively. Both systems displayed similar behavior, without significant changes. Therefore, the TiO_2_-F with silane suggests that silane does not modify hydrolytic degradation. However, the water absorption of the PLA/Wx/TiO_2_-F blends was minor at extended periods compared to that of PLA/Wx/TiO_2_-NF, suggesting that the amount of water absorbed by PLA/Wx/TiO_2_-F is the minimum required to produce this change.

### 3.3. Scanning Electron Microscopy

**Figure 5:**
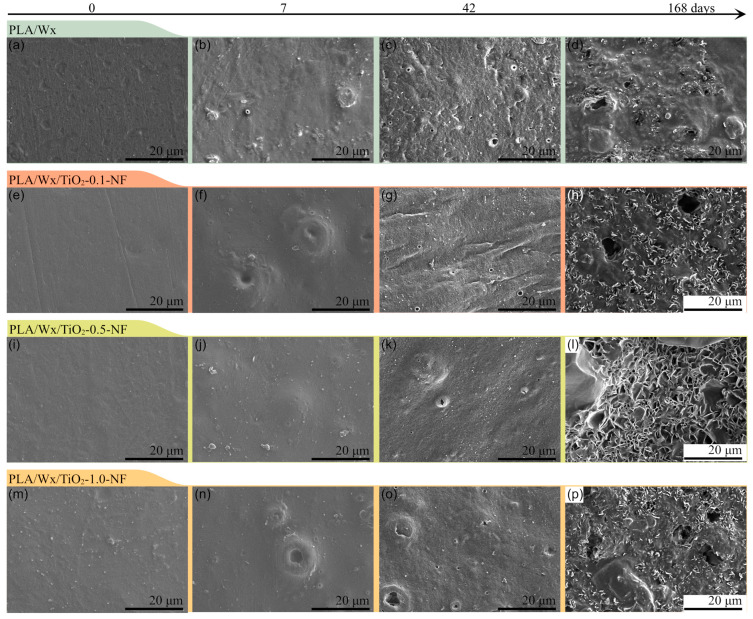
SEM micrographs for PLA/Wx/TiO_2_-NF blends vs. PLA/Wx at different times after the initiation of hydrolytic degradation. (**a**–**d**) PLA/Wx at 0, 7, 42, and 168 days, respectively; (**e**–**h**) PLA/Wx/TiO_2_-0.1-NF at 0, 7, 42, and 168 days, respectively; (**i**–**l**) PLA/Wx/TiO_2_-0.5-NF at 0, 7, 42, and 168 days, respectively; (**m**–**p**) PLA/Wx/TiO_2_-1.0-NF at 0, 7, 42, and 168 days, respectively.

**Figure 6:**
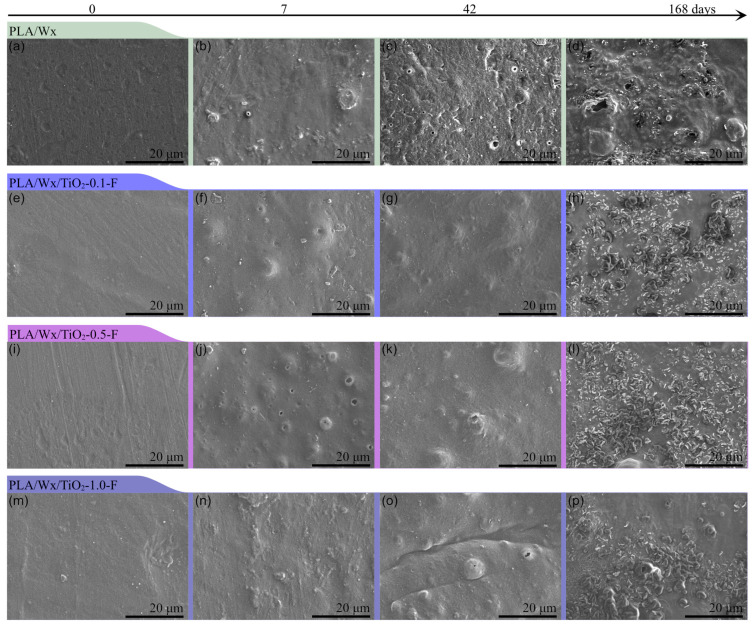
SEM micrographs for PLA/Wx/TiO_2_-F blends vs. PLA/Wx at different times after the initiation of hydrolytic degradation, particularly at 0, 7, 42, and 168 days. (**a**–**d**) PLA/Wx at 0, 7, 42, and 168 days, respectively; (**e**–**h**) PLA/Wx/TiO_2_-0.1-F at 0, 7, 42, and 168 days, respectively; (**i**–**l**) PLA/Wx/TiO_2_-0.5-F at 0, 7, 42, and 168 days, respectively; (**m**–**p**) PLA/Wx/TiO_2_-1.0-F at 0, 7, 42, and 168 days, respectively.

Figure 5 and Figure 6 illustrate micrographs at similar magnifications (20 µm), captured from the surface of the probes prior to hydrolytic degradation, as well as after 7, 42, and 168 days of immersion in water for probes made of the PLA/Wx/TiO_2_-NF and PLA/Wx/TiO_2_-F composites, versus PLA/Wx, respectively. Firstly, the surface of the PLA/Wx blend (Figure 5 or Figure 6a–d) initially appears slightly rough on day zero due to the Wx acting as a plasticizer, which affects the overall surface of the PLA/Wx blend. As hydrolytic degradation progresses, tiny bubbles begin to form, in particular, at 7 days. This finding could be explained by the fact that the water molecules come into contact with the PLA polymer chain and initiate the breakdown of its ester bonds, forming small gas pockets on the PLA/Wx blend’s surface [6]. After 42 days of degradation, this sample displays minor flaking. This change is a result of the ongoing hydrolysis process, where the polymer chains are being cleaved, weakening the material’s integrity and causing its surface to delaminate [35]. At 168 days, in addition to flaking, the presence of bubbles and holes is observed on a large part of the PLA/Wx blend’s surface.

Additionally, the addition of nanoparticles can affect hydrolytic degradation in multiple ways [6,36]. Factors such as the morphology, dispersion, and hydrophilic nature of nanoparticles can all play significant roles in determining how PLA degrades when exposed to moisture or water [6,36]. Some nanoparticles might hinder the degradation process by creating a barrier that reduces water penetration. In contrast, others might promote faster degradation due to their ability to attract water molecules or alter the polymer’s structure.

Regarding PLA/Wx/TiO_2_-NF blends (Figure 5), similar behavior is observed to that of the PLA/Wx blend at 0 and 7 days. At 42 days, these samples present considerable degradation compared to the PLA/Wx blend. This phenomenon can be clearly observed to be a result of the presence of large voids and more pronounced surface irregularities, suggesting the degradation and loss of polymer chains, as well as the erosion of the material’s surface over time [6]. These visual cues point to the material’s structural deterioration and the breakdown of its surface integrity. At 168 days, more voids and material losses are observed, especially for the PLA/Wx/TiO_2_-0.5-NF and PLA/Wx/TiO_2_-1.0-NF samples, indicating accelerated hydrolytic degradation. This result can be attributed to the presence of TiO_2_-NF, which enhances the hydrolytic degradation of PLA due to it acting as a nucleation site for the polymer chain. Previous studies have observed that the degradation efficiency of PLA was improved with the incorporation of TiO_2_ nanoparticles. For example, Luo et al. [19] conducted research on the long-term degradation of PLA composites containing different concentrations of TiO_2_ (ranging from 1 to 15 wt%) in a buffered solution maintained at 37 °C. They observed morphological changes in the material, which they attributed to modifications in the bulk erosion process. These changes were thought to be caused by uneven degradation occurring at the boundary between PLA and TiO_2_, which resulted in inhomogeneous deterioration at the PLA/TiO_2_ interface.

On the other hand, the PLA/Wx/TiO_2_-F (Figure 6) composites exhibit a similar behavior up until 42 days. The largest differences are observed at 168 days, with a decrease in the surface damage of the samples resulting from hydrolytic degradation. As noted in previous studies, the morphology, dispersion, and hydrophilic nature of nanoparticles can significantly influence the hydrolysis process. Some nanoparticles may hinder water penetration, while others might promote faster degradation by attracting water molecules and disrupting the polymer’s structure [6,36]. The decreased surface deterioration observed in the SEM micrographs for PLA/Wx/TiO_2_-F composites aligns with this theory, indicating that the TiO_2_-NF is facilitating the breakdown of the PLA matrix through a combination of hydrophilic interactions and accelerated hydrolysis.

**Figure 7:**
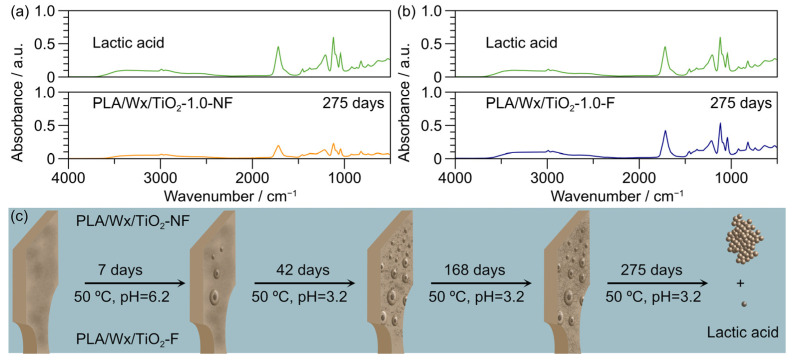
FTIR spectra of the hydrolytic degradation liquid at 7 and 275 days, compared against lactic acid. (**a**) PLA/Wx/TiO_2_-1.0-NF; (**b**) PLA/Wx/TiO_2_-1.0-NF; (**c**) evolution of the morphology of the samples under hydrolytic degradation.

During the 9-month hydrolysis process, all samples apparently retained their original shape; however, after 275 days, the samples completely lost their structure and could not be removed from the water for study. This unexpected behavior suggests bulk degradation, where the entire polymer breaks down simultaneously, but internal degradation progresses more quickly due to auto-acceleration effects [35]. Figure 7 displays the FTIR spectra of the hydrolytic degradation liquid at 275 days for the PLA/Wx/TiO_2_-1.0-NF (Figure 7a) and PLA/Wx/TiO_2_-1.0-F (Figure 7b) composites compared to lactic acid. The FTIR spectra of both composites are similar to that of lactic acid, suggesting the depolymerization or degradation of PLA to lactic acid. Figure 7c illustrates the evolution of this morphology for both composites. Overall, the hydrolytic degradation process, influenced by water penetration, nanoparticle dispersion, and interface effects, leads to surface roughening, bubble formation, flaking, and void creation, ultimately resulting in the breakdown of the material’s integrity over time [19,37].

### 3.4. Rheological Evaluation

**Figure 8:**
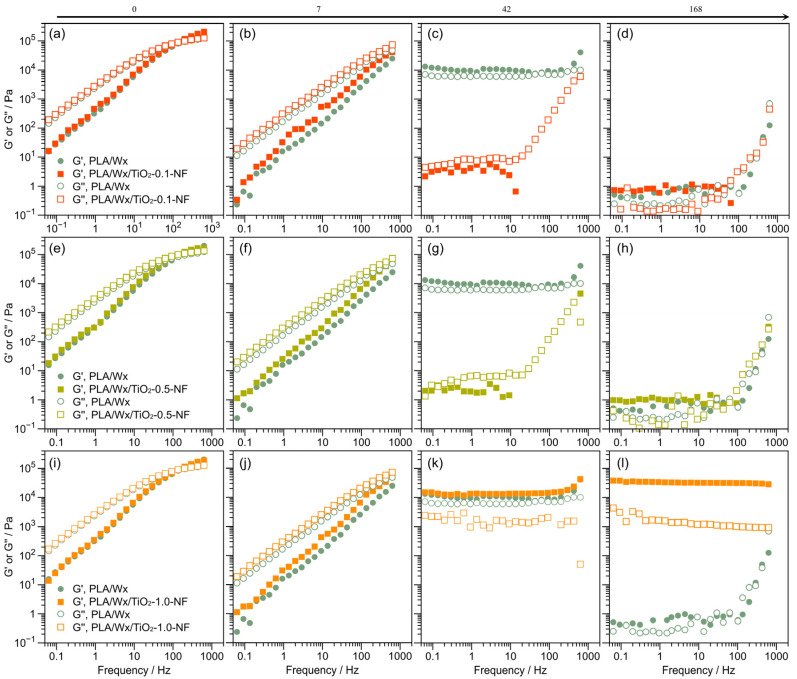
Rheological measurements with regard to the duration of hydrolytic degradation. (**a**) PLA/Wx/TiO_2_-0.1-NF at 0 days; (**b**) PLA/Wx/TiO_2_-0.1-NF at 7 days; (**c**) PLA/Wx/TiO_2_-0.1-NF at 42 days; (**d**) PLA/Wx/TiO_2_-0.1-NF at 168 days; (**e**) PLA/Wx/TiO_2_-0.5-NF at 0 days; (**f**) PLA/Wx/TiO_2_-0.5-NF at 7 days; (**g**) PLA/Wx/TiO_2_-0.5-NF at 42 days; (**h**) PLA/Wx/TiO_2_-0.5-NF at 168 days; (**i**) PLA/Wx/TiO_2_-1.0-NF at 0 days; (**j**) PLA/Wx/TiO_2_-1.0-NF at 7 days; (**k**) PLA/Wx/TiO_2_-1.0-NF at 42 days; (**l**) PLA/Wx/TiO_2_-1.0-NF at 168 days.

The linear viscoelastic properties of the blends were investigated through a dynamic frequency sweep. Figure 8 illustrates the storage (G′) and loss modulus (G″) for PLA/Wx/TiO_2_-0.1-NF (Figure 8a–d), PLA/Wx/TiO_2_-0.5-NF (Figure 8e–h), and PLA/Wx/TiO_2_-1.0-NF (Figure 8i–l) versus PLA/Wx after hydrolytic degradation for 0, 7, 42, and 168 days. Initially, all blends do not significantly change in terms of G′; see Figure 8a,e,i. However, in terms of their hydrolytic degradation at 7 days, all samples present liquid-like behavior, where their G″ exceeds G′; see Figure 8a,b,e,f,i,j. This is in opposition to extended periods of more than 7 days, where G′ displays higher values for PLA/Wx/TiO_2_-NF (all concentrations) and the PLA/Wx reference than G″, i.e., solid-like behavior; see Figure 8c,d,g,h,k,l. At 42 days, the PLA/Wx presents a behavior that is independent of frequency for G′ and G″. This finding could be explained as being due to degradation having begun in the PLA/Wx matrix, resulting in a slightly less rigid structure. In contrast, in the blends, the inclusion of the TiO_2_-NF or TiO_2_-F nanoparticles provides mechanical support, leading to a structure with higher G′ values. In the case of the PLA/Wx/TiO_2_-NF blend, at 42 days, this behavior was observed only at a TiO_2_-NF concentration of 1.0 wt %. This solid-like behavior could be attributed to the interaction between TiO_2_-NF and the polymer chains [38,39]. On day 168, the samples with lower concentrations of TiO_2_-NF nanoparticles (0.1 and 0.5 wt %) exhibit the same rheological behavior as PLA/Wx. However, the PLA/Wx/TiO_2_-1.0-NF sample maintains high values of G′, around 3 × 10^4^ Pa, and a solid-like behavior in the analyzed frequency range.

**Figure 9:**
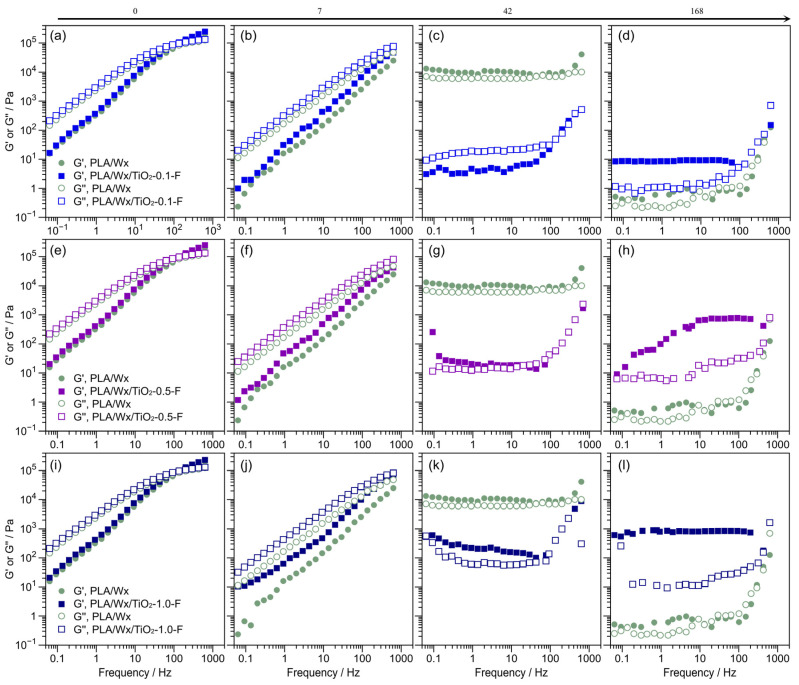
Rheological measurements with regard to the duration of hydrolytic degradation. (**a**) PLA/Wx/TiO_2_-0.1-F at 0 days; (**b**) PLA/Wx/TiO_2_-0.1-F at 7 days; (**c**) PLA/Wx/TiO_2_-0.1-F at 42 days; (**d**) PLA/Wx/TiO_2_-0.1-F at 168 days; (**e**) PLA/Wx/TiO_2_-0.5-F at 0 days; (**f**) PLA/Wx/TiO_2_-0.5-F at 7 days; (**g**) PLA/Wx/TiO_2_-0.5-F at 42 days; (**h**) PLA/Wx/TiO_2_-0.5-F at 168 days; (**i**) PLA/Wx/TiO_2_-1.0-F at 0 days; (**j**) PLA/Wx/TiO_2_-1.0-F at 7 days; (**k**) PLA/Wx/TiO_2_-1.0-F at 42 days; (**l**) PLA/Wx/TiO_2_-1.0-F at 168 days.

Figure 9 illustrates the storage (G′) and loss moduli (G″) for PLA/Wx/TiO_2_-0.1-F (Figure 9a–d), PLA/Wx/TiO_2_-0.5-F (Figure 9e–h), and PLA/Wx/TiO_2_-1.0-F (Figure 9i–l) versus PLA/Wx after hydrolytic degradation for 0, 7, 42, and 168 days. Compared to the PLA/Wx/TiO_2_-NF blends, the PLA/Wx/TiO_2_-F blends reveal identical behavior after 0, 7, and 42 days. Nevertheless, a different behavior was observed after 168 days. In addition, the G′ for PLA/Wx/TiO_2_-F was higher than that for PLA/Wx/TiO_2_-NF, except for the PLA/Wx/TiO_2_-1.0-F blend. This finding may be explained by the fact that the TiO_2_-F nanoparticles generate interactions with the degraded chains of the PLA [6,36].

### 3.5. Assay of Antibacterial Activity

**Figure 10:**
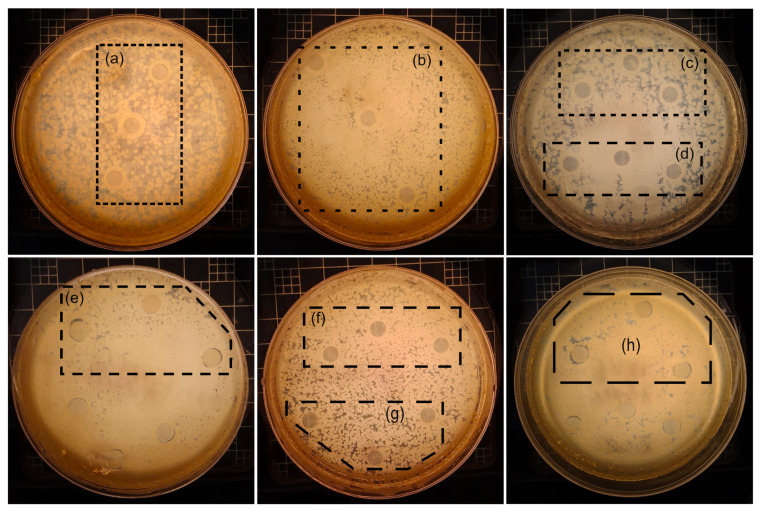
Images of the antibacterial tests of the samples: (**a**) PLA/Wx, *E. coli* at 24 h; (**b**) PLA/Wx, *S. aureus* at 24 h; (**c**) PLA/Wx/TiO_2_-0.1-NF, *E. coli* at 48 h; (**d**) PLA/Wx/TiO_2_-0.1-F, *E. coli* at 48 h; (**e**) PLA/Wx/TiO_2_-1.0-F, *E. coli* at 48 h; (**f**) PLA/Wx/TiO_2_-0.1-NF, *S. aureus* at 48 h; (**g**) PLA/Wx/TiO_2_-0.1-F, *S. aureus* at 48 h; and (**h**) PLA/Wx/TiO_2_-1.0-F, *S. aureus* at 48 h.

The antibacterial efficiency of PLA/Wx, PLA/Wx/TiO_2_-0.1-NF, and PLA/Wx/TiO_2_-0.1-F was assessed by testing them against both *E. coli* (Gram-negative) and *S. aureus* (Gram-positive) bacteria. Table 2 and Figure 10 display the results of the antibacterial experiment. In the case of the PLA/Wx film (Figure 10a,b), no antibacterial activity was observed, as no inhibition halo was formed. Additionally, the presence of a biofilm (bacterial layer) on and around its surface was observed, suggesting that this material failed to prevent the growth or proliferation of *E. coli* or *S. aureus*. On the other hand, the results for the PLA/Wx/TiO_2_-0.1-NF and PLA/Wx/TiO_2_-0.1-F films (Figure 10c,d,f,g) show a bacteriostatic effect against both bacteria. Although a biofilm formed around these materials, it did not develop directly on their surfaces, indicating that these materials inhibited bacterial growth and reproduction without necessarily eliminating the bacteria. This behavior suggests that the materials interfere with bacterial metabolism, limiting the development of bacteria without causing death [40]. The observed bacteriostatic effect is valuable for applications where bacterial proliferation needs to be controlled without completely eradicating all microorganisms. Materials like these help prevent infections and control bacterial spread in various areas, such as medical products, contact surfaces, and food preservation [41]. However, while they limit bacterial growth, they do not eliminate the bacteria. This highlights the importance of incorporating additional antibacterial properties into the materials used in the food industry to optimize their effectiveness in preventing contamination and microbial proliferation.

Furthermore, the high concentrations of TiO_2_-NF or TiO_2_-F in the PLA/Wx/TiO_2_-0.5-NF, PLA/Wx/TiO_2_-0.5-F, PLA/Wx/TiO_2_-1.0-NF, and PLA/Wx/TiO_2_-1.0-F films showed an inhibition halo, indicative of antibacterial activity. However, no significant differences were observed between these films, suggesting that chemical functionalization had no noticeable impact on these films’ antibacterial performance. This result is consistent with Črešnar et al. [42], who evaluated the antibacterial activity of TiO_2_ nanoparticles embedded in a PLA matrix at concentrations of 0.5, 1.0, and 2.5 wt%. They found that TiO_2_ nanoparticles exhibited considerably higher antibacterial activity against *E. coli* compared to *S. aureus*. The observed antibacterial activity may be attributed to the free radicals and reactive oxygen species generated by the TiO_2_ nanoparticles. When interacting with bacteria, these reactive oxygen species can cause direct damage to cell membrane components, disrupt membrane integrity, and, in some cases, penetrate the bacterial membrane. This process can destroy bacterial cells, promoting cell death [43].

Several nanoparticles with antibacterial properties have been synthesized and characterized for use in the production of single-use packaging materials. These include silver, zinc oxide, copper, copper oxide, gold, magnesium oxide, and others. These nanoparticles are effective in overcoming the problem of bacterial resistance, as they can eliminate more bacteria than traditional antibiotics. However, there are key differences between these nanoparticles and TiO_2_. In particular, TiO_2_ stands out for its low toxicity and high stability. It is an attractive option for applications where safety and durability are prioritized, such as developing materials for food packaging and other industrial uses [44,45].

### 3.6. Evaluation of Oxygen Gas Transmission Rate

Oxygen permeability is influenced by several factors that are intrinsic properties of the polymer and its blend. Additionally, the arrangement and packaging of the polymer molecules play a significant role in the permeation of oxygen through the material. The structural properties of the polymer, e.g., its amorphous or crystalline phase and how tightly the polymer chains are packed, can also affect its permeability to gases, e.g., oxygen [7]. Table 3 displays the oxygen permeation of the PLA/Wx, PLA/Wx/TiO_2_-NF, and PLA/Wx/TiO_2_-F blends. It is observed that regardless of the use of TiO_2_-NF or TiO_2_-F, these films’ oxygen permeation improves considerably compared to the PLA/Wx reference. In the case of the PLA/Wx/TiO_2_-NF samples, the oxygen permeation was lower because these particles have diameters of around 11–37 nm. Their inclusion could have disrupted the spaces between polymer chains to a lesser extent than the TiO_2_-F nanoparticles. Meanwhile, the findings when TiO_2_-F nanoparticles were employed in the formulation suggest an agglomeration of TiO_2_-F during the melt blending, as seen in Figure S1. In addition, this disruption increases when a higher amount of TiO_2_-NF nanoparticles is added, leading to greater oxygen permeation.

On the other hand, TiO_2_-F allows acceptable oxygen permeability. With the addition of 0.1 and 0.5 TiO_2_-F wt% to the PLA/Wx matrix, the amount of oxygen that permeated through the films was so substantial that it saturated the detectors of the equipment, making it impossible to register their oxygen permeation values. This result can be attributed to the agglomeration of TiO_2_-F during the melt blending and the creation of significant polymer chain disruption, through which an enormous amount of oxygen can permeate. Nonetheless, when the presence of these particles was increased to 1.0 wt%, their larger size could provide excellent packaging for the polymer chains, thereby reducing gas permeation, but not at the level registered by the PLA/Wx reference.

### 3.7. Film Transparency

The transparency, as a function of transmittance, from 200 to 900 nm of the PLA/Wx/TiO_2_-NF and PLA/Wx/TiO_2_-F films versus PLA/Wx films is shown in Figure S7. Table 4 illustrates the values of transmittance recorded in terms of optical transparency at 600 nm. For PLA/Wx/TiO_2_-NF and PLA/Wx/TiO_2_-F, their transmittance decreased in the visible region with respect to that of PLA/Wx. This decline is particularly noticeable in the samples containing 0.5% and 1% wt of TiO_2_-F. In the visible region, from 600 to 900 nm, PLA/Wx has a transmittance of about 94%. In contrast, the transparency of PLA/Wx/TiO_2_-1.0-F is about 86%. However, in the UV medium- and low-energy intervals, their transparency diminishes drastically with the addition of TiO_2_-NF and TiO_2_-F. The same behavior has been observed previously [47]. Other authors have reported transmittance values of around 40% at 340 nm with the addition of 1 wt% of TiO_2_ to a PLA matrix. Meanwhile, in our work, with identical TiO_2_ concentrations and wavelengths, the transmittance was around 54% and 41% for PLA/Wx/TiO_2_-1.0-NF and PLA/Wx/TiO_2_-1.0-F, respectively. At 300 nm, PLA/Wx’s transmittance corresponds to 70%. In contrast, with the addition of 0.5% TiO_2_-F and 1% TiO_2_-F, its transmittance is decreased to 23 and 22%, respectively, which represents a reduction in the UV transmittance of around 67% when compared with the reference. As reported before, in other PLA/nanoparticle systems, the smaller the transmittance, the more significant the absorption [31]. Finally, a comparison with our findings reveals that these composites show values similar to the ones reported previously, e.g., for LDPE, PHBV, PLLA, and PVC; see Table 4.

## 4. Conclusions

In this study, composites containing polylactic acid (PLA)/natural wax (Wx)/non-functionalized TiO_2_ nanoparticles (TiO_2_-NF) and PLA/Wx/TiO_2_ nanoparticles functionalized with triethoxysilane (TiO_2_-F) were systematically investigated. Their hydrolytic degradation, antimicrobial properties, and optical transparency were analyzed in detail.

The hydrolytic degradation behavior of these materials was systematically monitored over time, revealing the significant effects of the TiO_2_ nanoparticles on both the degradation process and the antibacterial properties of the PLA/Wx matrix. The results indicate that both TiO_2_-NF and TiO_2_-F nanoparticles notably influenced the degradation of PLA/Wx blends, with PLA/Wx/TiO_2_-NF composites exhibiting a substantial weight loss compared to the reference PLA/Wx sample. This enhanced degradation is likely attributed to increased liquid penetration as TiO_2_-NF particles detached from the polymer matrix. SEM analysis further corroborated these findings, showing the pronounced surface degradation, larger voids, and surface irregularities in the PLA/Wx/TiO_2_-NF samples, particularly after 42 days of hydrolytic exposure. The incorporation of TiO_2_-NF and TiO_2_-F into the PLA/Wx blends significantly altered their hydrolytic degradation behavior, e.g., TiO_2_-NF accelerates the degradation process.

Antibacterial testing revealed that TiO_2_-NF and TiO_2_-F exhibited bacteriostatic effects at 0.1 wt%, inhibiting the growth of *E. coli* without killing the bacteria. Notably, TiO_2_-F demonstrated stronger antibacterial activity against *E. coli*, while silane functionalization had minimal effect on the activity of the nanoparticles against *S. aureus*. This finding is significant, suggesting the potential use of these composites as antimicrobial materials where the growth of harmful bacteria needs to be controlled.

Regarding oxygen permeability, the PLA/Wx/TiO_2_-F composites displayed saturation at lower concentrations (0.1 and 0.5 wt%). In contrast, the PLA/Wx/TiO_2_-NF composites exhibited oxygen permeability similar to that of other polymers, such as LDPE films. Likewise, the optical transparency determined by UV-Vis spectroscopy showed transmittance values from 86% to 94% for all formulations. These values are comparable to those of other polymers such as PHVB, LDPE, PVC, and PLLA.

In conclusion, these findings provide valuable insights into the potential for tailoring the biodegradation rate of PLA-based materials, particularly for applications requiring controlled degradation, antibacterial activity, and UV protection.

## Figures and Tables

**Table 1:** Formulations of polylactic acid (PLA)/natural wax (Wx)/non-functionalized TiO_2_ nanoparticles (TiO_2_-NF) or TiO_2_ nanoparticles functionalized with triethoxysilane (TiO_2_-F).

Sample	PLA/wt%	Beeswax/wt%	Cocoa Wax/wt%	TiO_2_-NF/wt%	TiO_2_-F/wt%
PLA/Wx	85.0	7.5	7.5	0	0
PLA/Wx/TiO_2_-0.1-NF	84.9	7.5	7.5	0.1	0
PLA/Wx/TiO_2_-0.5-NF	84.9	7.5	7.5	0.5	0
PLA/Wx/TiO_2_-1.0-NF	84.5	7.5	7.5	1.0	0
PLA/Wx/TiO_2_-0.1-F	84.5	7.5	7.5	0	0.1
PLA/Wx/TiO_2_-0.5-F	84.0	7.5	7.5	0	0.5
PLA/Wx/TiO_2_-1.0-F	84.0	7.5	7.5	0	1.0

**Table 2:** Assay of inhibition zones of PLA/Wx/TiO_2_-NF and PLA/Wx/TiO_2_-F versus PLA/Wx.

Sample	Conc. TiO_2_/wt%	*E. coli* (Inhibition Zone (mm))		*S. aureus* (Inhibition Zone (mm))	
		24 h	48 h	24 h	48 h
PLA/Wx	0.0	No antibacterial activity		No antibacterial activity	
PLA/Wx/TiO_2_-0.1-NF	0.1	Bacteriostatic	Bacteriostatic	Bacteriostatic	Bacteriostatic
PLA/Wx/TiO_2_-0.5-NF	0.5	8.83 ± 0.40	9.44 ± 0.85	8.67 ± 0.33	8.74 ± 0.14
PLA/Wx/TiO_2_-1.0-NF	1.0	8.74 ± 0.60	8.93 ± 0.36	8.62 ± 0.18	8.59 ± 0.58
PLA/Wx/TiO_2_-0.1-F	0.1	Bacteriostatic	Bacteriostatic	Bacteriostatic	Bacteriostatic
PLA/Wx/TiO_2_-0.5-F	0.5	9.12 ± 0.52	9.29 ± 0.34	8.64 ± 0.40	8.50 ± 0.17
PLA/Wx/TiO_2_-1.0-F	1.0	9.16 ± 0.27	9.47 ± 0.42	8.40 ± 0.47	8.67 ± 0.72

**Table 3:** Oxygen permeability of PLA/Wx/TiO_2_-NF and PLA/Wx/TiO_2_-1.0-F versus PLA/Wx.

Sample	Oxygen Permeability (cm^3^ cm/cm^2^ s Pa)	Reference
HDPE films	4.56 × 10^−14^–8.33 × 10^−14^	[46]
LDPE films	18.2 × 10^−14^–23.9 × 10^−14^	[46]
Oriented PET	0.12 × 10^−14^	[46]
PLA, PLA/1% TiO_2_	2.0 × 10^−14^, 1.8 × 10^−14^	[7]
PLA/Wx	2.5 × 10^−14^	In this work
PLA/Wx/TiO_2_-0.1-NF	23.0 × 10^−14^	
PLA/Wx/TiO2-0.5-NF	23.7 × 10^−14^	
PLA/Wx/TiO_2_-1.0-NF	33.4 × 10^−14^	
PLA/Wx/TiO_2_-1.0-F	25.9 × 10^−14^	

**Table 4:** Optical transparency of PLA/Wx/TiO_2_-NF and PLA/Wx/TiO_2_-1.0-F versus PLA/Wx.

Sample	Transmittance_600 nm_/%	Reference
PHBV (Poly(3-hydroxybutyrate-*co*-3-hydroxyvalerate))	85	[48]
LDPE (low-density polyethylene)	87	[48,49]
PVC (Polyvinyl chloride)	90	[48]
PLLA (Poly-L-lactide)	89	[48]
PLA/Wx	93	In this work
PLA/Wx/TiO_2_-0.1-NF	94	
PLA/Wx/TiO_2_-0.5-NF	95	
PLA/Wx/TiO_2_-1.0-NF	92	
PLA/Wx/TiO_2_-0.1-F	96	
PLA/Wx/TiO_2_-0.5-F	93	
PLA/Wx/TiO_2_-1.0-F	86	

## Data Availability

The original contributions presented in this study are included in the article/Supplementary Materials. Further inquiries can be directed to the corresponding authors.

## Supplementary Materials

The following supporting information can be downloaded at https://www.mdpi.com/article/10.3390/polym17050685/s1, Figure S1: particle size distribution of non-functionalized titanium dioxide nanoparticles (TiO_2_-NF); Figure S2: energy-dispersive X-ray spectroscopy (EDX) of TiO_2_-NF; Figure S3: X-ray spectrum of TiO_2_-NF; Figure S4: particle size distribution of titanium dioxide nanoparticles functionalized with triethoxysilane (TiO_2_-F); Figure S5: energy-dispersive X-ray spectroscopy (EDX) of TiO_2_-F; Figure S6: FTIR spectra of polylactic acid (PLA), beeswax, cocoa, PLA/Wx (beeswax and cocoa), PLA/Wx/TiO_2_-1.0-F, and PLA/Wx/TiO_2_-1.0-NF; Figure S7: Transmittance percentage of the films. (a) PLA/Wx/TiO_2_-0.1-NF, PLA/Wx/TiO_2_-0.5-NF, PLA/Wx/TiO_2_-1.0-NF, and PLA/Wx; (b) PLA/Wx/TiO_2_-0.1-F, PLA/Wx/TiO2-0.5-F, PLA/Wx/TiO_2_-1.0-F, and PLA/Wx.

## Abbreviations

The following abbreviations are used in this manuscript:

PLA	Polylactic acid
Wx	Natural waxes
TiO2-NF	Non-functionalized TiO2 nanoparticles
TiO2-F	Functionalized TiO2 nanoparticles
