# Supplementary material for: Unraveling of Poly(lactic acid) (PLA)/Natural Wax/Titanium Dioxide Nanoparticle Composites for Disposable Plastic Applications
Source: Polymers (Basel). 2025 Mar 4;17(5):685. doi: 10.3390/polym17050685 (PMC11902720; doi:10.3390/polym17050685)
Supplement: Supplementary file 1 [file polymers-17-00685-s001.zip › polymers-3475510-supplementary.docx]

Unraveling of Poly(lactic acid) (PLA) / Natural Wax Composites with Titanium Dioxide Nanoparticles for Disposable Plastic Applications.

Jacqueline Bocarando-Chacón ^1^, Iván Alziri Estrada-Moreno ^2^, Imelda Olivas-Armendáriz ^3^, Alejandro Vega Rios ^4,*^ and Mónica Elvira Mendoza-Duarte ^4,^*

^1^ Universidad Tecnológica de Querétaro. Av. Pie de la Cuesta 2501, Nacional, Qro, Santiago de Querétaro, 76148, México; jacqueline.bocarando@uteq.edu.mx

^2^ Secretaría de Ciencia, Humanidades, Tecnología e Innovación (SECIHTI) - CIMAV; ivan.estrada@cimav.edu.mx

^3^  Institute of Engineering and Technology, Autonomous University of the City of Juárez (UACJ), Ave. Del Charro 450 Norte, Ciudad Juárez, 32310, Mexico; iolivas@uacj.mx

^4^  Centro de Investigación en Materiales Avanzados, SC, Av. Miguel de Cervantes #120, Chihuahua 31136, Mexico.

***** Correspondence: alejandro.vega@cimav.edu.mx (A.V.-R.); monica.mendoza@cimav.edu.mx (M.E.M.-D.)


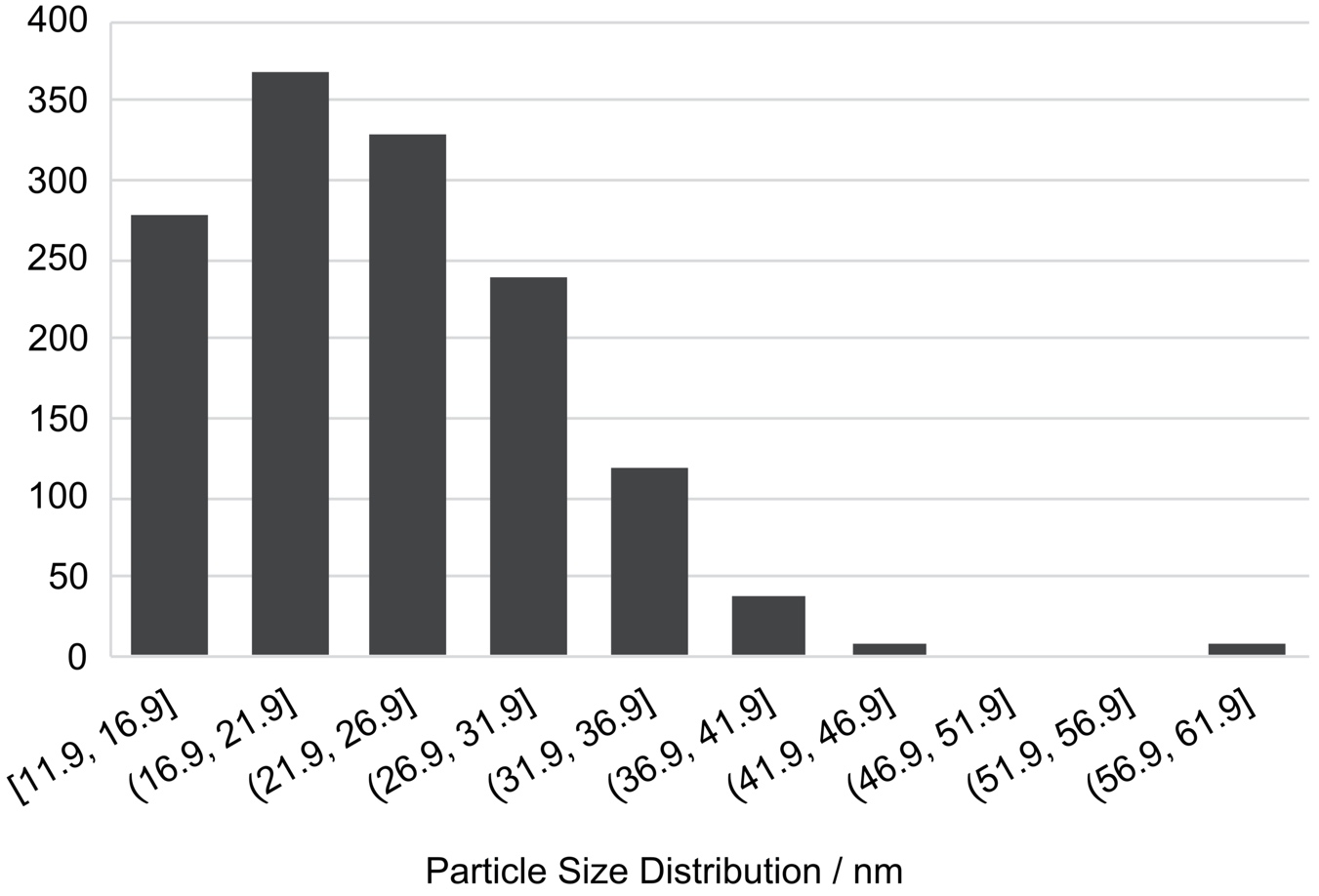


**Figure S1.** Particle size distribution of non-functionalized titanium dioxide nanoparticles (TiO_2_-NF).


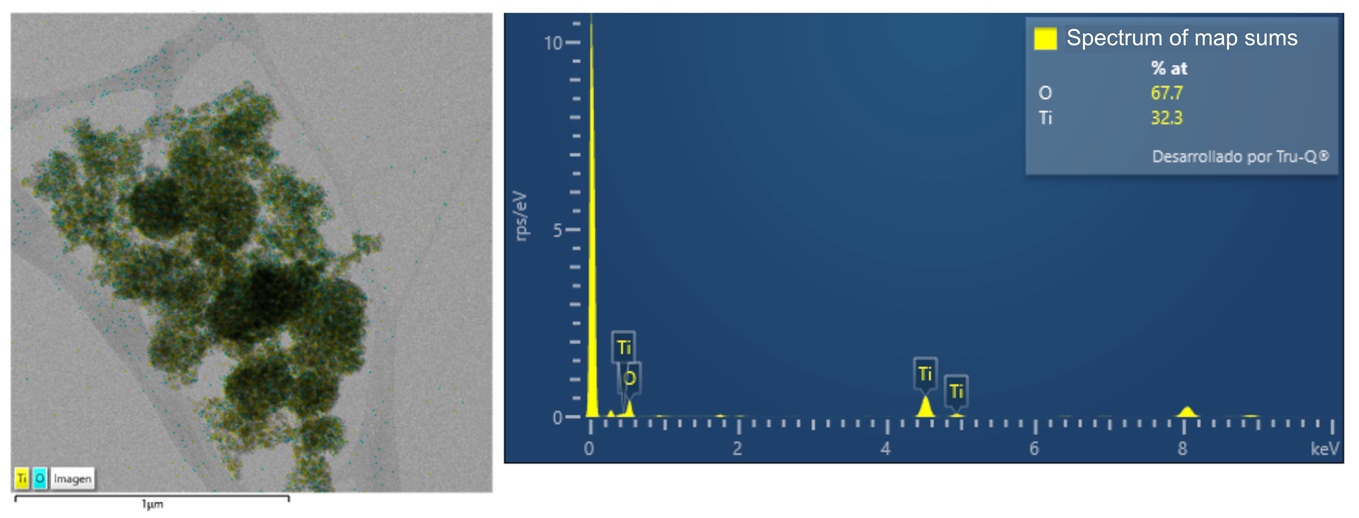


**Figure S2.** Energy dispersive X-ray spectroscopy (EDX) of TiO_2_-NF.


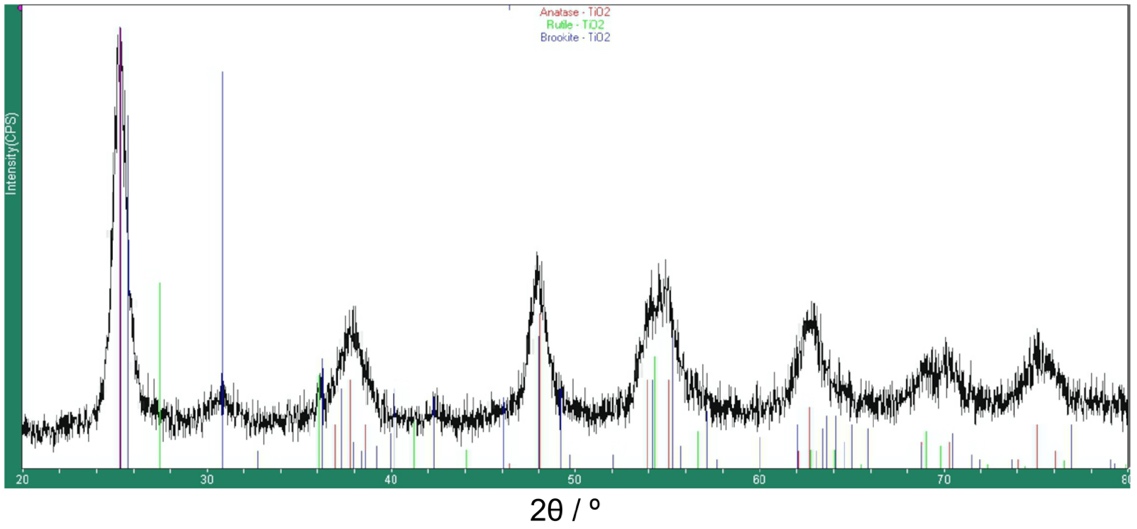


**Figure S3.** X-ray spectrum of TiO_2_-NF.


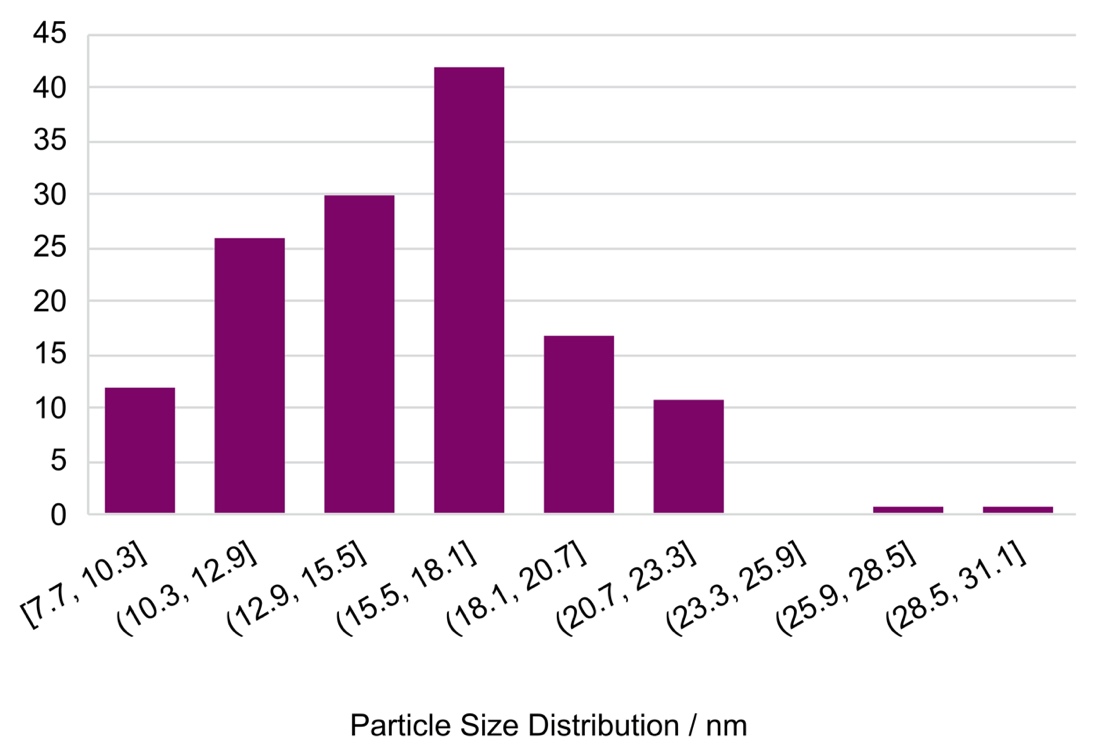


**Figure S4.** Particle size distribution of titanium dioxide nanoparticles functionalized with triethoxysilane (TiO_2_-F).


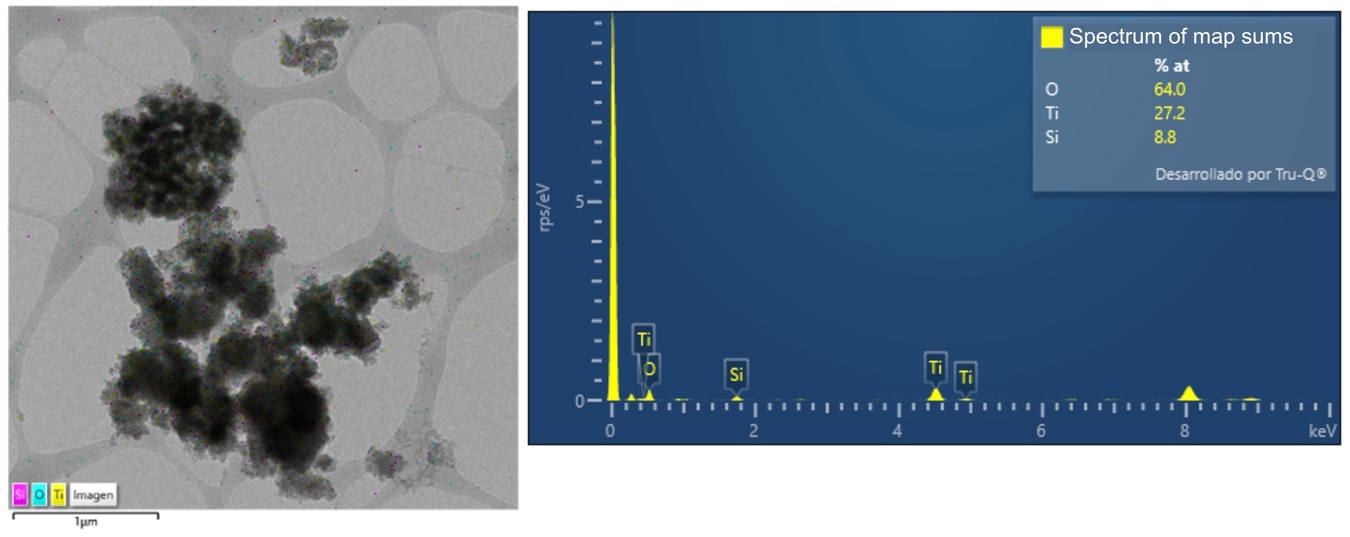


**Figure S5.** Energy dispersive X-ray spectroscopy (EDX) of TiO_2_-F.


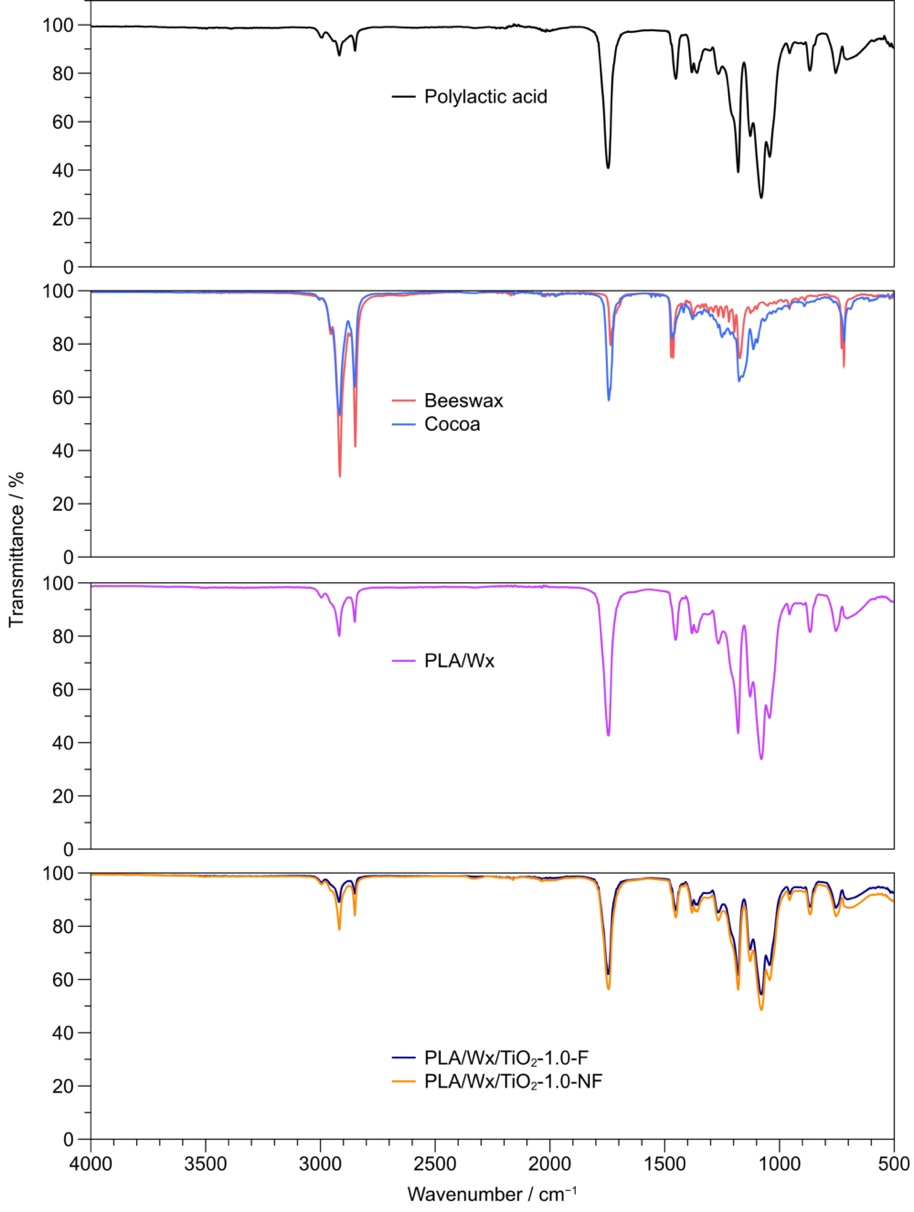


**Figure S6.** FTIR spectra of polylactic acid (PLA), Beeswax, Cocoa, PLA/Wx (Beeswax and Cocoa), PLA/Wx/TiO_2_-1.0-F and PLA/Wx/TiO_2_-1.0-NF.


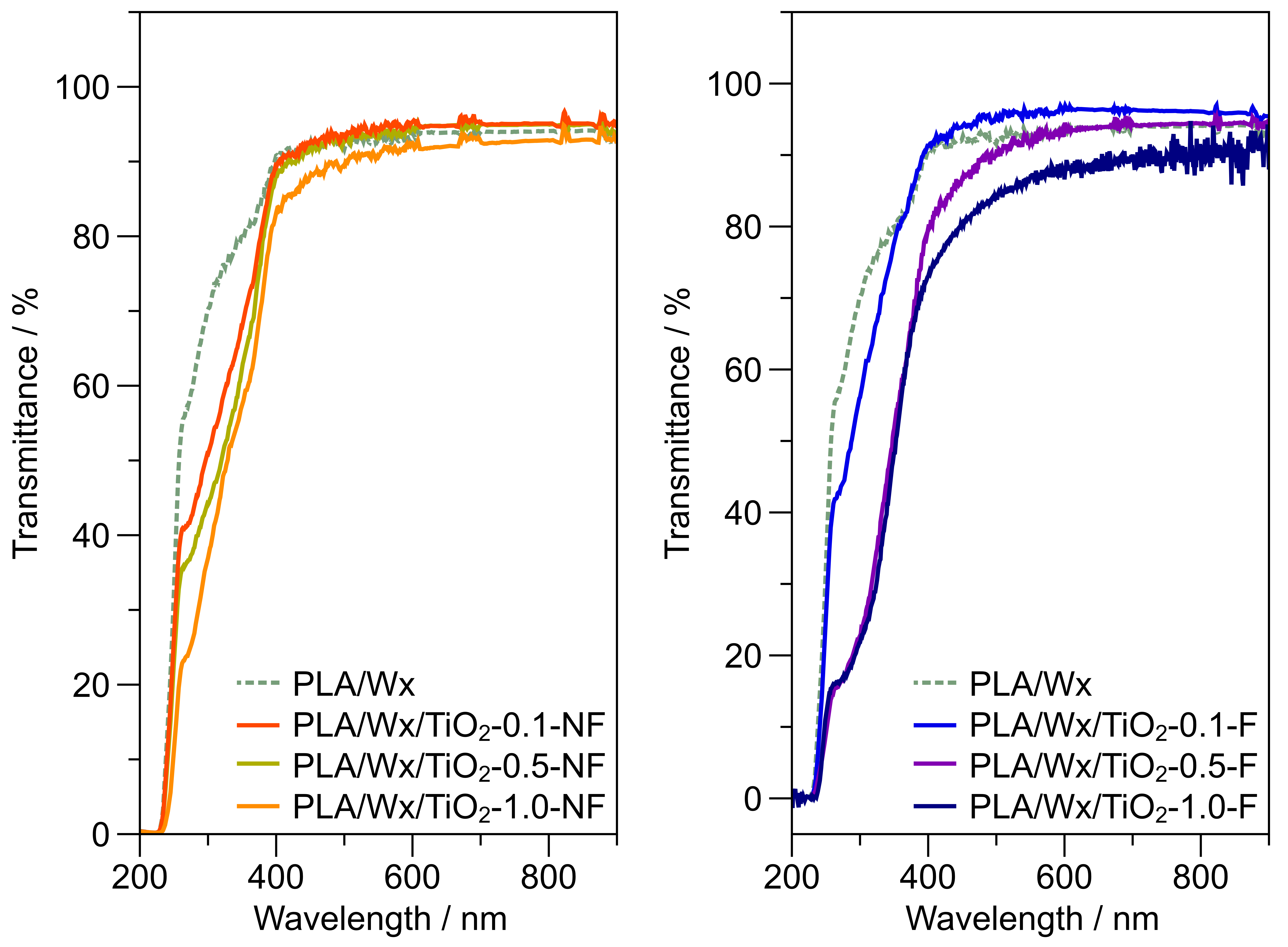


**Figure S7.** Transmittance percentage of the films. (**a**) PLA/Wx/TiO_2_-0.1-NF, PLA/Wx/TiO_2_-0.5-NF, PLA/Wx/TiO_2_-1.0-NF and PLA/Wx; (**b**) PLA/Wx/TiO_2_-0.1-F, PLA/Wx/TiO_2_-0.5-F, PLA/Wx/TiO_2_-1.0-F and PLA/Wx.
